# Breadth of Antibodies to *Plasmodium falciparum* Variant Surface Antigens Is Associated With Immunity in a Controlled Human Malaria Infection Study

**DOI:** 10.3389/fimmu.2022.894770

**Published:** 2022-05-30

**Authors:** Hannah W. Kimingi, Ann W. Kinyua, Nicole A. Achieng, Kennedy M. Wambui, Shaban Mwangi, Roselyne Nguti, Cheryl A. Kivisi, Anja T. R. Jensen, Philip Bejon, Melisa C. Kapulu, Abdirahman I. Abdi, Samson M. Kinyanjui, CHMI-SIKA Study Team, Abdirahman I Abdi

**Affiliations:** ^1^ Kenya Medical Research Institute (KEMRI) Wellcome Trust Research Programme, Kilifi, Kenya; ^2^ Department of Biological Sciences, Pwani University, Kilifi, Kenya; ^3^ School of Public Health, Faculty of Health Sciences, University of the Witwatersrand, Johannesburg, South Africa; ^4^ Pwani University Bioscience Research Centre, Pwani University, Kilifi, Kenya; ^5^ Centre for Medical Parasitology, Department of Immunology and Microbiology, Faculty of Health and Medical Sciences, University of Copenhagen, Copenhagen, Denmark; ^6^ Centre for Tropical Medicine and Global Health, Nuffield Department of Medicine, University Oxford, Oxford, United Kingdom; ^7^ School of Business Studies, Strathmore University, Nairobi, Kenya

**Keywords:** malaria, *Plasmodium falciparum*, CHMI, variant surface antigens, anti-VSA antibodies, antibody breadth, PfEMP1, ICAM1

## Abstract

**Background:**

*Plasmodium falciparum* variant surface antigens (VSAs) contribute to malaria pathogenesis by mediating cytoadhesion of infected red blood cells to the microvasculature endothelium. In this study, we investigated the association between anti-VSA antibodies and clinical outcome in a controlled human malaria infection (CHMI) study.

**Method:**

We used flow cytometry and ELISA to measure levels of IgG antibodies to VSAs of five heterologous and one homologous *P. falciparum* parasite isolates, and to two PfEMP1 DBLβ domains in blood samples collected a day before the challenge and 14 days after infection. We also measured the ability of an individual’s plasma to inhibit the interaction between PfEMP1 and ICAM1 using competition ELISA. We then assessed the association between the antibody levels, function, and CHMI defined clinical outcome during a 21-day follow-up period post infection using Cox proportional hazards regression.

**Results:**

Antibody levels to the individual isolate VSAs, or to two ICAM1-binding DBLβ domains of PfEMP1, were not associated with a significantly reduced risk of developing parasitemia or of meeting treatment criteria after the challenge after adjusting for exposure. However, anti-VSA antibody breadth (i.e., cumulative response to all the isolates) was a significant predictor of reduced risk of requiring treatment [HR 0.23 (0.10-0.50) p= 0.0002].

**Conclusion:**

The breadth of IgG antibodies to VSAs, but not to individual isolate VSAs, is associated with protection in CHMI.

## Introduction

The number of malaria cases has declined substantially over the last few decades (1, 2), but 627 000 attributable deaths globally in the year 2020 (1) still marks malaria out as a major health concern especially in children under 5 years of age in sub-Saharan Africa. The looming threat of mosquito resistance to insecticides (3) and changes in feeding behavior (4), together with the emergence of malaria parasite resistance to drugs including artemisinin based combinations (5), has placed a strong focus on vaccines as potentially the most effective ways of eradicating malaria. The most advanced malaria vaccine to date, RTS,S, confers partial and transient immunity to pre-erythrocytic malaria parasites (6–8), thus pointing to the need to develop vaccines that induce more potent and long-lasting immunity. However, this is hampered by the complexity of the malaria parasite’s life cycle, the variant and polymorphic nature of many malaria antigens, and the lack of clear correlates of immunity to malaria (9–12). Nonetheless, immuno-epidemiological studies showing that humans acquire immunity through repeated exposure to malaria (13–16) and passive antibody transfer experiments (17, 18) provide the incentive for the continued search for more efficacious malaria vaccines against blood-stage parasites.

Among the proteins considered potential candidates for malaria vaccine are the variant surface antigens (VSAs) displayed on the surface of red blood cells infected by the mature blood stage of malaria parasites (19–26). *P. falciparum* erythrocyte membrane protein 1 (PfEMP1), one of the best studied VSAs, plays an important role in malaria pathogenesis by mediating the cytoadhesion and sequestration of infected red blood cells to the endothelium of host blood vessels as a way of escaping immune clearance. This adhesion, which is mediated by host receptors such as ICAM1, EPCR and CD36, results in vascular occlusion and inflammation, which are hallmarks of severe malaria (27–31). The wide diversity of VSAs poses a challenge in their inclusion in malaria vaccines. However, several longitudinal studies have shown that antibodies to VSAs reduce the risk of being reinfected by a variant recognized by those antibodies, but not necessarily to other variants, but there are also longitudinal studies that suggest that a degree of cross-variant immunity also exists (24, 25, 32), thus justifying the continued interest in understanding the role of anti-VSA antibodies in immunity to malaria.

A complexity in studying immunity to malaria is in distinguishing between immunological responses that are simply markers of exposure and those that are causally linked to immunity, as both increase concurrently with repeated exposure to malaria. In addition, exposure to infected mosquito bites in the field is heterogeneous, making it difficult to distinguish between non-exposure and genuine immunity as the reason for apparent protection in field-based immunology studies (33). Controlled human malaria infection study (CHMI) overcomes these challenges by ensuring homogenous exposure with subsequent stringent monitoring of parasitemia and symptoms. We used CHMI studies in Kenya to further explore the role of anti-VSA antibodies in immunity to malaria. We sought to determine whether levels and function of anti-VSA antibodies were associated with better clinical outcome among the CHMI participants.

## Methods

### Study Design and Population

This study was nested in a larger controlled human malaria infection (CHMI) study conducted at KEMRI-Wellcome Trust Research Programme (KWTRP) as described previously (34). Briefly, healthy adults were recruited for CHMI from Ahero (high malaria endemicity), Kilifi South (high malaria endemicity) and Kilifi North (low malaria endemicity) locations in Kenya. Following initial screening, 161 volunteers were administered with 3.2×10^3^ NF54 *P. falciparum* sporozoites and monitored for parasite growth and clinical outcome for a period of 21 days. Parasite growth was monitored using qPCR and individuals who reached a parasite density of 500 parasites/µL or developed fever were given anti-malarial drugs, while the rest were all treated on day 22 (34). The blood samples collected from the participants were processed to separate plasma and cells and stored at -80°C until use. For this study, we used plasma collected a day before (C-1) and 14 days after the challenge (C+14).

### Parasite Isolates

Six *P. falciparum* isolates were used for this study, two laboratory-adapted cultures (SAO75 and A4U) and four heterologous *ex-vivo* isolates (6454, 19462, 19477, and NF54). The *ex-vivo* parasite isolates (except NF54) were obtained from patients admitted to the High Dependency Unit at Kilifi County Hospital with severe malaria and cultured to mature trophozoites stage before being frozen. The laboratory adapted isolates were retrieved from liquid nitrogen storage, grown to mature trophozoite and then frozen. The NF54 parasites isolated from the study participants and expanded in culture appeared not to express VSAs as we observed poor antibody recognition by serum from highly immune positive controls as well as study participant’s plasma samples **(** **Supplementary Figure 1** **)**. This is in line with previous studies that have shown that continuous culture decreases *var* gene transcription and protein expression (35, 36). We therefore produced an NF54 culture selected on ICAM1 and CD36 to enhance VSA expression, referred to hereafter as ‘NF54(ICAM1/CD36)’. All the isolates were cultured in RPMI 1640 medium (Sigma) supplemented with 2mM L-glutamine, 37.5mM HEPES, 20mM glucose, 50µg/mL sodium hypoxanthine, 25µg/mL gentamicin and 0.5% albumax (all from Gibco) (37) and cryopreserved in glycerolyte (38).

### Detection of Anti-VSA Antibodies by Flow Cytometry

The frozen trophozoites were thawed as previously described (38). A 10% suspension of the thawed infected red blood cells pellet was prepared using 1XPBS. 2.5µL of the 10% pellet suspension was added into 8.5µL of 1XPBS/0.5%BSA then stained with 10µg/µL Ethidium Bromide (EtBr). The suspension was incubated in a U-bottomed 96 well plate with 1µL of test plasma and malaria naïve control plasma for 30 minutes at room temperature to allow for antigen-antibody binding. The cells were then washed three times using 1XPBS/0.5%BSA by centrifugation at 110×g for 3 minutes. 50µL of fluorescein isothiocyanate (FITC) conjugated sheep anti-human IgG (Binding Site, UK) was added at 1:50 dilution and this was incubated for 30 minutes in the dark, then washed 3 times with 1XPBS/0.5%BSA by centrifugation at 100×g for 3 minutes. The pellet was then suspended in 200µL of 1XPBS. 100µL of the re-suspended pellet was further diluted with 400µL of 1XPBS in a FACS tube to bring the pellet volume to 0.05µL and 1000 trophozoite-infected erythrocytes were acquired from each tube on a FACSCanto flow cytometer (Beckman Coulter, UK).

### Gating Strategy

Data obtained from FACSCanto flow cytometer was analyzed using FlowJo version 10. Ethidium Bromide staining was used to distinguish between infected and uninfected red blood cells, while the intensity of the FITC signal was considered a proxy of the level of anti-VSA IgG antibodies. Background staining of uninfected red blood cells was corrected by subtracting the median fluorescent intensity (MdFI) of the uninfected red blood cells from the MdFI of the infected red blood cells. To correct for non-specific antibody binding, the background adjusted MdFI of 13 malaria-naive European plasma was subtracted from the MdFI test plasma.

### Expression of Recombinant Protein Domains

The ICAM1-binding DBLβ domains of PF3D7_0425800 (previous ID PFD1235w) and PF3D7_1150400 (previous ID PF11_0521) were expressed as HIS-tagged proteins in *Escherichia coli* Shuffle C3030 cells (New England BioLabs) and purified by immobilized metal ion affinity chromatography using HisTrap HP 1-mL columns (GE Healthcare) as described previously (28, 39). Recombinant Fc-tagged ICAM-1 was expressed in HEK293 cells and purified as described previously (40).

### Detection of DBLβ Reactive Antibodies

ELISA was used to analyze IgG reactivity to the above PfEMP1 DBLβ domains. Briefly, Immunolon plates (Thermo Scientific) were coated with 2.5µg/mL of the recombinant proteins PF3D7_1150400 (PF11_0521) and PF3D7_0425800 (PFD1235w) diluted in ELISA carbonate coating buffer (Thermo Scientific)and incubated overnight at 4°C. The plates were then washed in PBST (1X PBS with 0.05% Tween 20) followed by blocking with PBST and 1% skimmed milk for 1h at room temperature. After washing with PBST, plasma samples (1:100 in blocking buffer) were added and incubated for 1h at room temperature. Binding was detected using rabbit anti-human IgG-HRP (Dako, 1:5000 in blocking buffer). Malaria naïve plasma samples were used as negative controls.

### Detection of Antibodies to *P. falciparum* Schizont Extract

CHMI plasma samples collected a day before the challenge (C-1) were analyzed for presence of antibodies against *P. falciparum* schizont extract using ELISA as previously published (41). Briefly, *P. falciparum* 3D7 parasites were cultured to schizont stage. The harvested culture was sonicated, followed by a series of freeze-thaw cycles to prepare the schizont extract. Different dilutions of the schizont extract were coated on ELISA plates and a pool of plasma from hyper immune individuals was used to detect antibodies against the extract and determine the optimal dilution of the extract to use for subsequent assays. Standard ELISA protocol was followed to measure antibodies against the schizont extract in the CHMI plasma samples.

### Inhibition of Binding of PfEMP1 to ICAM1

To measure the ability of an individual’s plasma to inhibit the interaction between the recombinant PfEMP1 DBLβ domain (PF3D7_1150400/PF11_0521) and ICAM1, we used competition ELISA. First, Immunolon plates (Thermo Scientific) were coated with 5µg/mL of PF3D7_1150400 recombinant protein and incubated overnight at 4°C. The plates were then washed and blocked with PBST and 1% skimmed milk for 1h at room temperature. After washing, ICAM1–Fc (5µg/mL) and plasma (1:10) were added simultaneously and incubated for 1h at room temperature (42). The plates were then washed four times with PBST. Bound ICAM1 was detected by mouse anti-human ICAM-1 (PE anti-human CD54, cat.no 353106, BioLegend, 1:500) followed by goat anti-mouse IgG-HRP, (Dako, 1:5000). Plasma samples from malaria naïve individuals from the UK were used as negative controls.

### Statistical Analysis

Data analysis was carried out using R version 4.0.5. For all tests, *P* values of <0.05 were considered significant. Graphs were generated using GraphPad Prism 8. The adjusted MdFI was log transformed in R and normality of data was checked using Shapiro test. Cox proportional hazard risk model was used to assess the association between anti-VSA antibody levels and two definitions of CHMI outcomes: 1) Time to establishment of a PCR-detectable infection and 2) time to requiring treatment during follow up.

To test if CHMI outcome was associated with the breadth of anti-VSA antibodies across the whole panel of test isolates rather than responses to individual isolates we developed an antibody response breadth score for each participant. This was done by assigning the participant’s response to each isolate a score of 0, 1, 2 or 3 depending on whether the response was in the lowest, middle, or upper quartile of the responses to that isolate respectively. The total score across the six isolates (minimum = 0, maximum = 18) was taken as the individual’s anti-VSA antibodies breadth and was further 3 equal categorized as “low” (total score 0-6), “medium” (total score 7-12) or “high” (total score 13-18). The association between the breadth categories and CHMI outcome was assessed using cox-proportional hazard model.

Additionally, the association between antibodies to two PfEMP1 DBLβ domains (PF3D7_1150400/PF11_0521 and PF3D7_0425800/PFD1235w) and CHMI outcome was also assessed. The antibody-mediated inhibition of ICAM1 binding of the DBLβ domain was analyzed from two independent experiments carried out in duplicates and the association with CHMI outcomes assessed using Cox proportional hazard risk model.

In all cases a univariable and subsequent multivariable analysis with further stepwise regression was carried out and the results are reported as “unadjusted” and “adjusted”.

To test if CHMI outcome was associated with changes in antibody levels between day C-1 and day 14 after challenge, Mann-Whitney U test was used to assess for significant difference between the treated and untreated groups as well as the PCR positive and PCR negative groups.

## Results

### CHMI Outcome

Of the 161 participants enrolled for CHMI, 19 were excluded from the final analysis due to infection with *P. falciparum* isolates other than NF54 during follow-up or presence of anti-malarial drug in their plasma as previously described (41, 43). The 142 participants included in the final analysis were grouped as either untreated PCR negative (n=33), untreated PCR positive (n=53), treated non-febrile (n=30), or treated febrile (n=26) based on parasite growth monitored by qPCR or development of fever during follow-up. Further details of the outcomes are described elsewhere (43).

### Anti-VSA Antibodies Response to a Panel of Parasite Isolates

**Figure 1:**
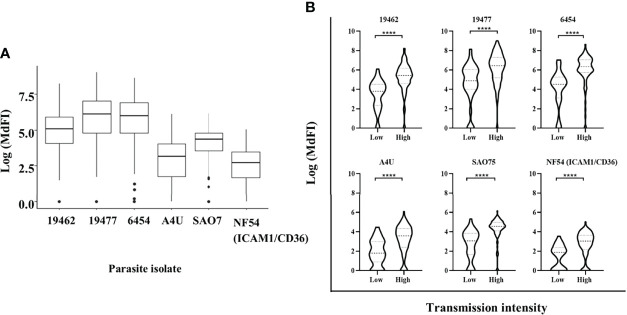
Anti-VSA antibodies response and malaria endemicity. **(A)** The median and interquartile range of anti-VSA antibodies levels (expressed as log median fluorescent intensity) against the panel of isolates. **(B)** Comparison of anti-VSA IgGs (expressed as log median fluorescent intensity) among individuals from low and high malaria transmission areas. (**** - P-value = <0.0001).

All the participants showed varied levels of anti-VSA antibodies to all the test isolates. The *ex-vivo* isolates were better recognized with highest levels of antibodies being against isolate 19477 and 6454 and the lowest against NF54 (ICAM1/CD36) (**Figure 1A**). Participants from Kilifi South and Ahero, which are high malaria transmission areas, had higher levels of antibodies compared to those from Kilifi North which is a low malaria transmission area **(** **Figure 1B** **)**.

### Anti-VSA Antibodies and Risk of Developing PCR-Detectable Infection During CHMI

Antibody levels to each of the parasite isolates except NF54(ICAM1/CD36), overall antibody breadth, binding inhibition levels and anti-schizont antibodies were all significantly associated with reduced risk of the endpoint of a PCR detectable infection in univariable analysis **(** **Table 1** **).** However, there was marked collinearity of the different responses with varying degrees of positive cross-correlations **(** **Supplementary Figure 2** **)**. Antibodies to the specific parasite isolates were not independently associated with reduced risk of acquiring PCR detectable infection in a multivariable analysis model that adjusted for antibodies to schizont extract as indicated in **Table 1**.

**Table 1:** Univariable and multivariable cox regression models for risk of developing detectable parasitemia after the challenge.

Variable	Univariable		Multivariable (all variables)		Multivariable (restricted)	
	HR (95% CI)	P value	HR (95% CI)	P value	HR (95% CI)	P value
Anti-19462	0.74 (0.62-0.90)	0.0019	0.81 (0.56-1.18)	0.28	NA	NA
Anti-19477	0.80 (0.65-0.99)	0.04	1.23 (0.84-1.79)	0.28	NA	NA
Anti-6454	0.72 (0.60-0.87)	0.0005	0.83 (0.57-1.21)	0.34	0.92 (0.68-1.24)	0.59
Anti-A4U	0.80 (0.67-0.96)	0.01	0.89 (0.67-1.19)	0.44	NA	NA
Anti-SAO75	0.72 (0.61-0.85)	0.0002	0.87 (0.64-1.19)	0.38	0.89 (0.66-1.20)	0.44
Anti-NF54 (ICAM1/CD36)	0.87 (0.72-1.04)	0.12	NA	NA	NA	NA
Antibody breadth	0.75 (0.61-0.92)	0.01	1.22 (0.74-2.02)	0.43	NA	NA
Anti-PF3D7_1150400	0.96 (0.84-1.10)	0.58	NA	NA	NA	NA
Anti-PF3D7_0425800	1.03 (0.88-1.21)	0.70	NA	NA	NA	NA
Binding inhibition	0.79 (0.64-0.99)	0.04	0.89 (0.70-1.14)	0.36	NA	NA
Anti-Schizont	0.57 (0.44-0.74)	<0.0001	0.70 (0.51-0.96)	0.03	0.67 (0.49-0.92)	0.01

Multivariable (restricted): Stepwise regression including the factors indicated in the table.

NA, Indicates factors not included in the model.

### Anti-VSA Antibodies and Risk of Meeting a Threshold for Anti-Malarial Treatment During CHMI

Similarly, univariable analysis showed that antibody levels to each of the parasite isolates, overall antibody breadth, binding inhibition levels and anti-schizont antibodies were all associated with a significant reduction in the risk of meeting a threshold for malaria treatment during follow-up (**Table 2**). However, only breadth of antibody responses and anti-schizont antibodies were significant predictors of reduced risk of reaching a threshold for anti-malarial treatment on multivariable regression analysis (**Table 2** and **Supplementary Table 1** **)**. Further analysis showed that the untreated group had significantly higher levels of anti-VSA antibodies compared to those who required treatment **(** **Supplementary Figure 3** **).** Similarly, the untreated group showed a higher binding inhibition ability compared to the treated group (**(** **Supplementary Figure 4** **).**

**Table 2:** Univariable and multivariable cox regression models for risk of meeting a threshold for treatment after the challenge.

Variable	Univariable		Multivariable (all variables)		Multivariable (restricted)	
	HR (95% CI)	P value	HR (95%CI)	P value	HR (95%CI)	P value
Anti-19462	0.57 (0.46-0.70)	<0.0001	0.65 (0.39-1.09)	0.10	NA	NA
Anti-19477	0.58 (0.47-0.72)	<0.0001	1.52 (0.89-2.59)	0.12	NA	NA
Anti-6454	0.51 (0.42-0.63)	<0.0001	0.88 (0.49-1.60)	0.68	NA	NA
Anti-A4U	0.62 (0.48-0.79)	0.0001	1.63 (1.06-2.53)	0.03	NA	NA
Anti-SAO75	0.54 (0.45-0.66)	<0.0001	1.00 (0.67-1.49)	0.99	NA	NA
Anti-NF54 (ICAM1/CD36)	0.61 (0.48-0.78)	0.0001	1.81 (1.07-3.03)	0.03	NA	NA
Antibody breadth	0.33 (0.24-0.46)	<0.0001	0.23 (0.10-0.50)	0.0002	0.46 (0.32-0.67)	<0.0001
Anti-PF3D7_1150400	0.83 (0.71-0.96)	0.01	1.27 (0.69-2.32)	0.44	NA	NA
Anti-PF3D7_0425800	0.98 (0.79-1.22)	0.85	NA	NA	NA	NA
Binding inhibition	0.58 (0.40-0.83)	0.0028	0.71 (0.43-1.16)	0.17	NA	NA
Anti-Schizont	0.13 (0.21-0.34)	<0.0001	0.31 (0.17-0.55)	0.0001	0.40 (0.25-0.64)	0.0001

Multivariable (restricted): Stepwise regression including the factors indicated in the table.

NA, Indicates factors not included in the model.

**Figure 2:**
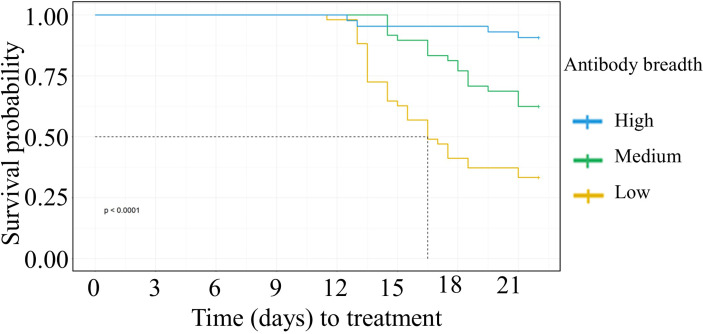
Kaplan Meier survival analysis of time to treatment stratified by individuals’ breadth of anti-VSA antibodies at day C-1. The dotted line denotes the median survival time where the survival probability is below 50%. Medium breadth score: HR= 0.38(95%CI 0.21-0.67, p=0.0008). High breadth score: HR= (0.08(95%CI 0.03-0.24,p=<0.0001).

We categorized the participant’s anti-VSA antibody breadth into three categories, low, medium, or high. Having either medium or high antibody breadth was associated with significantly longer time to treatment compared to having low antibody breadth **(** **Figure 2** **).** In addition, individuals who remained PCR negative and those that did not require treatment during follow up had significantly higher antibody breadth compared to those that required treatment **(** **Supplementary Figures 5A, B** **).**

### Boosting of Heterologous Anti-VSA Antibodies After NF54 Challenge

**Figure 3:**
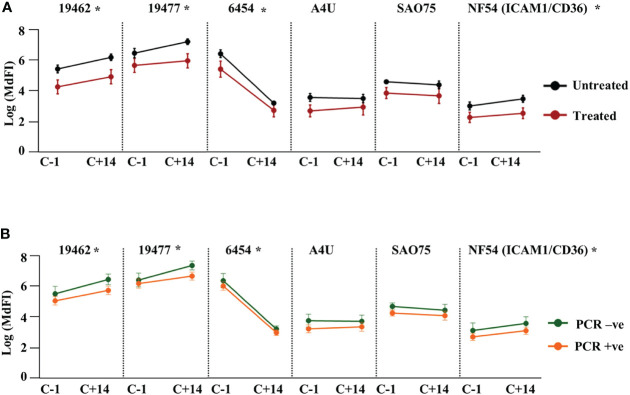
Change in heterologous anti-VSA antibodies levels after the challenge. Anti-VSA antibodies to the test isolates a day before and on day 14 after the challenge stratified by: **(A)** treatment outcome and **(B)** parasitaemia (*- P<0.05).

Next, we assessed the effect of NF54 challenge on the levels of anti-VSA antibodies to the test isolates. Comparing the response one day prior to challenge with responses 14 days (C+14) after the challenge, we observed significant boosting of antibodies against two field isolates 19462, 19477 and the NF54 (ICAM1/CD36) lab isolate. By contrast, the antibody levels to isolate 6454 dropped significantly by C+14, while there were no significant changes in the levels of antibody to the remaining two parasite isolates **(** **Supplementary Figure 6** **).** We then stratified the participants by outcome and compared the changes in antibody responses between the groups. The untreated group showed a significantly larger drop in antibodies to isolate 6454 compared to the treated group while there was no difference in the change between the two groups for all the other isolates. The same pattern of change was observed whether the outcome considered was requiring treatment or establishment of a PCR detectable infection **(** **Figures 3A, B** respectively).

## Discussion

In this study, we assessed the role of anti-VSA antibodies in immunity against malaria in CHMI participants using three approaches. We looked at the association between either having anti-VSA antibodies to individual isolates or the cumulative antibodies, or having antibodies to specific PfEMP1 domains, or having functional anti-VSA and CHMI outcome defined by either establishment of a PCR detectable infection or meeting the threshold for requiring treatment.

The notable finding in this study is that having a wide breadth of anti-VSA antibodies to a panel of randomly selected isolates is associated with a reduced risk of meeting the threshold for treatment in a controlled human malaria infection with isolate NF54. Although anti-VSA antibodies to individual isolates and to two PfEMP1 DBLβ domains were significantly associated with reduced risk of treatment in a univariable analysis, the association was not observed in a multivariable analysis. On the other hand, none of the anti-VSA antibodies or related parameters were associated with immunity against establishing a PCR-detectable infection following a multivariable analysis.

There are two potential explanations for these findings: The first is that all the apparent associations between anti-VSA antibodies and CHMI outcomes are confounded, and high anti-VSA antibodies are just markers of immunity mediated by other mechanisms which increase co-incidentally with exposure. To account for this possibility, we used a multivariable analysis model that included antibodies to schizont extract. The extract represents potentially all blood-stage malaria parasite antigens and therefore should correct for confounding protection by mechanisms targeted at other antigens that were not specifically tested here. The fact that having a wide antibody breadth remained significantly associated with reduced risk of reaching a threshold for treatment even after adjusting for schizont extract suggest that cumulative anti-VSA antibodies, by their own right, can provide protection against high parasitemia and/or symptoms in CHMI.

The lack of association between any of the parameters relating to anti-VSA antibodies and protection against establishment of a PCR-detectable parasitemia is consistent with observations in immuno-epidemiological studies suggesting some individuals acquire a form of immunity that protect from clinical symptoms but not infection (44, 45). On the other hand, the observation that specific anti-VSA responses to individual isolates were not associated with protection, but that breadth of anti-VSA antibodies to a randomly selected panel of isolates protects against high parasitemia and symptoms is interesting, given that prior work reports variant specificity of anti-VSA antibodies (25, 46). It is possible that in addition to the predominantly variant specific anti-VSA antibodies responses, malaria infections also generates a small amount of partially cross-reactive anti-VSA antibodies (47). With repeated infections, the partially cross-reactive antibodies accumulate sufficiently to provide significant protection against symptomatic infection by a wide range of variants including NF54.

We further explored the possibility that the heterologous anti-VSA antibodies were cross-reactive with the NF54 specific anti-VSA antibodies by looking at the changes in the levels of the antibodies 14 days after challenge. Significant boosting was apparent for antibodies to two of the clinical isolates (19477 and 19462). There was also a slight boost in the levels of anti-NF54 (ICAM1/CD36), but a significant drop was observed in the case of anti-6454 antibodies. There was no change in the level of antibodies to the lab isolates. The observed changes were similar whether all participants were considered together or stratified by CHMI outcome. The boosting of antibodies to field isolates by the challenge infection suggest that there may be some level of cross-reactivity with NF54. By extension, the lack of boosting of antibodies by the lab isolates may be reflecting the narrowness of the antibodies specific to these clonal isolates. However, the NF54 (ICAM1/CD36) parasites that we use have been selected *in vitro* for specific VSAs binding to ICAM1/CD36, and *in vivo* NF54 is likely to express a greater variety of VSAs. Furthermore, it is then perhaps not surprising that no single anti-VSA response predicts immunity, since individuals with a single anti-VSA specificity will result in parasite selection for alternative VSA expression. On the other hand, individuals with a broad range of anti-VSA responses are protected against parasite growth due to protection against cytoadherence. We speculate that protection against low-level parasitemia requires other immunological responses.

This study was limited by inability to measure the levels of anti-VSA antibodies to the actual infecting NF54 isolate. Parasites isolated from the participants appeared not to express VSAs upon culturing and were not recognised by antibodies from any of the participants. Altered or loss of expression of VSAs upon *in vitro* culturing is a commonly observed phenomenon (48). This is due to many factors, including use of Albumax instead of human serum to supplement the culture media which influences knobs and VSA expression (49). To circumvent this problem, we used NF54 that was selected to express VSAs binding to the endothelial receptors ICAM1 and CD36 by panning on recombinant ICAM1 and CD36 proteins and confirmed to select for expression of VSAs that bind to these proteins (data not shown). Although homologous to the isolate used to infect the CHMI participants, the selected isolate may represent only a portion of the full NF54 VSA repertoire. Second, recent findings have highlighted the importance of persistent IgM responses against blood stage antigens (50). In this study, we only measured IgG antibodies and we cannot make any conclusion regarding IgM antibodies to VSAs among the study participants. We therefore recommend further studies to examine the role of pre-existing anti-VSA IgM in parasite growth control and disease outcome in CHMI.

Previous longitudinal studies have shown that VSAs are associated with variant-specific immunity to malaria, but most of the studies are either in children or pregnant women (51–53). This study suggests that, despite VSA diversity, there is some level of cross-protection (albeit small) between variants. Understanding the mechanism behind this cross-protection, for example, if it is driven by conserved, but poorly immunogenic epitopes, could form the basis for further studies on VSA as potential vaccine candidates.

## Members of the CHMI-SIKA Study Team

Abdirahman I Abdi, KEMRI-Wellcome Trust Research Programme, Kilifi, Kenya; Yonas Abebe, Sanaria Inc., Rockville, MD, USA; Agnes Audi, Centre for Clinical Research, Kenya Medical Research Institute, Kisumu, Kenya; Philip Bejon, KEMRI-Wellcome Trust Research Programme, Kilifi, Kenya; Centre for Tropical Medicine and Global Health, Nuffield Department of Medicine, University Oxford, Oxford, UK; Peter Billingsley, Sanaria Inc., Rockville, MD, USA; Peter C Bull, Department of Pathology, University of Cambridge, Cambridge, UK; Primus Che Chi, KEMRI-Wellcome Trust Research Programme, Kilifi, Kenya; Zaydah de Laurent, KEMRI-Wellcome Trust Research Programme, Kilifi, Kenya; Susanne H Hodgson, The Jenner Institute, University of Oxford, Oxford, UK.; Stephen Hoffman, Sanaria Inc., Rockville, MD, USA; Eric James, Sanaria Inc., Rockville, MD, USA; Irene Jao, KEMRI-Wellcome Trust Research Programme, Kilifi, Kenya; Dorcas Kamuya, KEMRI-Wellcome Trust Research Programme, Kilifi, Kenya; Gathoni Kamuyu, KEMRI-Wellcome Trust Research Programme, Kilifi, Kenya; Silvia Kariuki, KEMRI-Wellcome Trust Research Programme, Kilifi, Kenya; Nelson Kibinge, KEMRI-Wellcome Trust Research Programme, Kilifi, Kenya; Sam Kinyanjui, KEMRI-Wellcome Trust Research Programme, Kilifi, Kenya; Centre for Clinical Research, Kenya Medical Research Institute, Kisumu, Kenya; Pwani University, P. O. Box 195-80108, Kilifi, Kenya; Cheryl Kivisi, Pwani University, P. O. Box 195-80108, Kilifi, Kenya; Nelly Koskei, Centre for Clinical Research, Kenya Medical Research Institute, Kisumu, Kenya; Mallika Imwong, Faculty of Tropical Medicine, Department of Molecular Tropical Medicine and Genetics, Mahidol University, Bangkok, Thailand; Brett Lowe, KEMRI-Wellcome Trust Research Programme, Kilifi, Kenya; Centre for Tropical Medicine and Global Health, Nuffield Department of Medicine, University Oxford, Oxford, UK; Johnstone Makale, KEMRI-Wellcome Trust Research Programme, Kilifi, Kenya; Kevin Marsh, KEMRI-Wellcome Trust Research Programme, Kilifi, Kenya; Centre for Tropical Medicine and Global Health, Nuffield Department of Medicine, University Oxford, Oxford, UK; Vicki Marsh, KEMRI-Wellcome Trust Research Programme, Kilifi, Kenya; Centre for Tropical Medicine and Global Health, Nuffield Department of Medicine, University Oxford, Oxford, UK; Khadija Said Mohammed, KEMRI-Wellcome Trust Research Programme, Kilifi, Kenya; Moses Mosobo, KEMRI-Wellcome Trust Research Programme, Kilifi, Kenya; Sean C Murphy, Departments of Laboratory Medicine and Microbiology, University of Washington, Seattle, Washington, USA; Jennifer Musyoki, KEMRI-Wellcome Trust Research Programme, Kilifi, Kenya; Michelle Muthui, KEMRI-Wellcome Trust Research Programme, Kilifi, Kenya; Jedidah Mwacharo, KEMRI-Wellcome Trust Research Programme, Kilifi, Kenya; Daniel Mwanga, KEMRI-Wellcome Trust Research Programme, Kilifi, Kenya; Joyce Mwongeli, KEMRI-Wellcome Trust Research Programme, Kilifi, Kenya; Francis Ndungu, KEMRI-Wellcome Trust Research Programme, Kilifi, Kenya; Maureen Njue, KEMRI-Wellcome Trust Research Programme, Kilifi, Kenya; George Nyangweso, KEMRI-Wellcome Trust Research Programme, Kilifi, Kenya; Domitila Kimani, KEMRI-Wellcome Trust Research Programme, Kilifi, Kenya; Joyce M. Ngoi, KEMRI-Wellcome Trust Research Programme, Kilifi, Kenya; Janet Musembi, KEMRI-Wellcome Trust Research Programme, Kilifi, Kenya; Omar Ngoto, KEMRI-Wellcome Trust Research Programme, Kilifi, Kenya; Edward Otieno, KEMRI-Wellcome Trust Research Programme, Kilifi, Kenya; Bernhards Ogutu, Centre for Clinical Research, Kenya Medical Research Institute, Kisumu, Kenya; Center for Research in Therapeutic Sciences, Strathmore University, Nairobi, Kenya; Fredrick Olewe, Centre for Clinical Research, Kenya Medical Research Institute, Kisumu, Kenya; James Oloo, Centre for Clinical Research, Kenya Medical Research Institute, Kisumu, Kenya; Donwilliams Omuoyo, KEMRI-Wellcome Trust Research Programme, Kilifi, Kenya; John Ongecha, Centre for Clinical Research, Kenya Medical Research Institute, Kisumu, Kenya; Martin O Ongas, Centre for Clinical Research, Kenya Medical Research Institute, Kisumu, Kenya; Center for Research in Therapeutic Sciences, Strathmore University, Nairobi, Kenya; Michael Ooko, KEMRI-Wellcome Trust Research Programme, Kilifi, Kenya; Jimmy Shangala, KEMRI-Wellcome Trust Research Programme, Kilifi, Kenya; Betty Kim Lee Sim, Centre for Tropical Medicine and Global Health, Nuffield Department of Medicine, University Oxford, Oxford, UK; Joel Tarning, Centre for Tropical Medicine and Global Health, Nuffield Department of Medicine, University Oxford, Oxford, UK; Mahidol-Oxford Tropical Medicine Research Unit, Mahidol University, Bangkok, Thailand; Juliana Wambua, KEMRI-Wellcome Trust Research Programme, Kilifi, Kenya; Thomas N Williams, KEMRI-Wellcome Trust Research Programme, Kilifi, Kenya; Department of Medicine, Imperial College, London, UK; Markus Winterberg, Centre for Tropical Medicine and Global Health, Nuffield Department of Medicine, University Oxford, Oxford, UK; Mahidol-Oxford Tropical Medicine Research Unit, Mahidol University, Bangkok, Thailand

## Data Availability Statement

The datasets presented in this study can be found in online repositories. The names of the repository/repositories and accession number(s) can be found below: Harvard Dataverse, URL: https://doi.org/10.7910/DVN/AYWX0Y.

## Ethics Statement

Ethical approval was obtained from KEMRI Scientific and Ethics Review Unit (KEMRI//SERU/CGMR-C/029/3190) and the University of Oxford Tropical Research Ethics Committee (OxTREC 2-16). The patients/participants provided their written informed consent to participate in this study.

## Author Contributions

SK, AA, and CK designed the study. HK, AK, KM, and SK analyzed the data. HK, AK, and SK and wrote the first draft of the manuscript. PB, MK, SK, AA, and CK edited the manuscript. HK, AK, RN, NA, and SM performed flow cytometry experiments. NA performed binding inhibition assays. AJ provided the PfEMP1 recombinant proteins and edited the manuscript. All authors contributed to interpretation of the analyses and revised the draft manuscript.

## Funding

This work was, in part, supported by the Tackling Infections to Benefit Africa (TIBA, NIHR Project no. 16/136/33) and in part, by The Wellcome Trust (grant no.107769/Z/10/Z) and the UK Foreign, Commonwealth and Development Office, with support from the Developing Excellence in Training Science and Leadership in Africa DELTAS Africa Initiative [DEL-15-003]. CAK was funded by the Wellcome Trust (grant 080883). ATRJ is supported by a Lundbeck Foundation grant (R313-2019-322). The views expressed in this publication are those of the author(s) and not necessarily those of NHS, NIHR, Wellcome Trust, Lundbeck Foundation, or the UK government.

## Conflict of Interest

The authors declare that the research was conducted in the absence of any commercial or financial relationships that could be construed as a potential conflict of interest.

## Publisher’s Note

All claims expressed in this article are solely those of the authors and do not necessarily represent those of their affiliated organizations, or those of the publisher, the editors and the reviewers. Any product that may be evaluated in this article, or claim that may be made by its manufacturer, is not guaranteed or endorsed by the publisher.
