# Supplementary material for: Breadth of Antibodies to Plasmodium falciparum Variant Surface Antigens Is Associated With Immunity in a Controlled Human Malaria Infection Study
Source: Front Immunol. 2022 May 30;13:894770. doi: 10.3389/fimmu.2022.894770 (PMC9195513; doi:10.3389/fimmu.2022.894770)
Supplement: Supplementary file 1 [file DataSheet_1.docx]

**SUPPLEMENTARY MATERIAL**

**
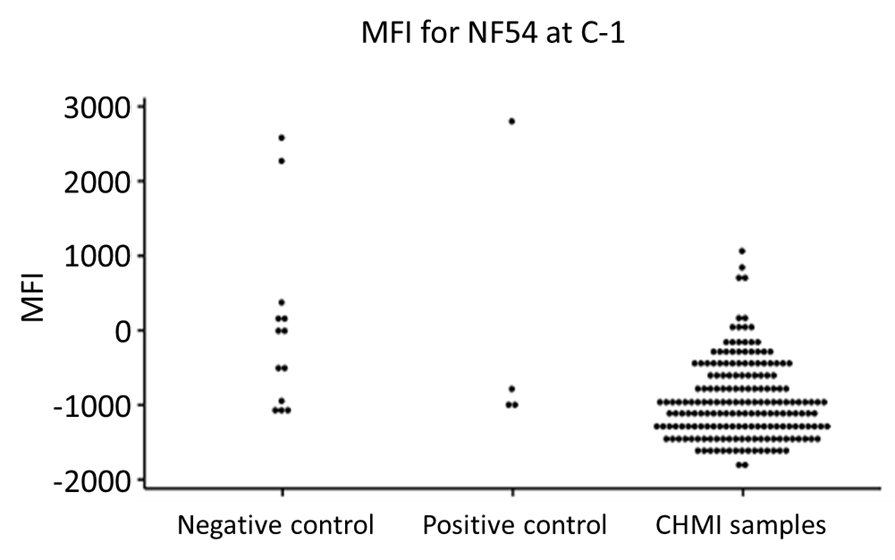
**

**Supplementary Figure 1: Antibody response to variant surface antigens expressed by the NF54 parasite isolate.** Anti-VSA antibodies levels are expressed as mean fluorescent intensity (MFI). Negative controls are plasma samples from malaria naïve individuals, positive controls are a pool of hyperimmune plasma samples collected from individuals residing in a high malaria endemic area, CHMI plasma samples were collected a day before the challenge.

**
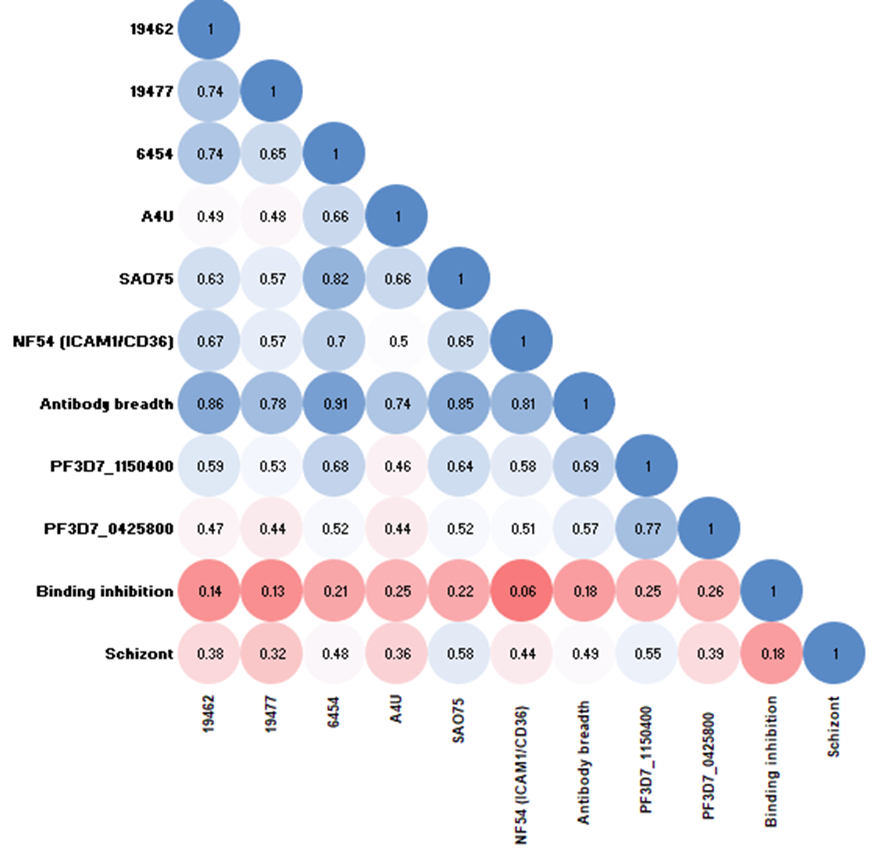
**

**Supplementary Figure 2:** Correlations between antibodies to VSAs expressed by red blood cells infected with the indicated *P. falciparum* isolates, antibody breadth, antibodies two PfEMP1 recombinant proteins, antibodies against *P. falciparum* schizont extract and binding inhibition functional assay. Shown are Spearman's rank correlation coefficients.

**Supplementary Table 1**: Univariable and multivariable cox regression models for risk of requiring treatment after the challenge

|  |  |  |  |  |  |  |  |  |
| --- | --- | --- | --- | --- | --- | --- | --- | --- |
|  | Univariable | | Multivariable (all variables) | | Multivariable (restricted) | | Multivariable (restricted) | |
| **Variable** | **HR (95% CI)** | **P value** | **HR (95%CI)** | **P value** | **HR (95%CI)** | **P value** | **HR (95%CI)** | **P value** |
| Anti-19462 | 0.57 (0.46-0.70) | <0.0001 | 0.65 (0.39-1.09) | 0.10 | 0.77 (0.50-1.16) | 0.21 | NA | NA |
| Anti-19477 | 0.58 (0.47-0.72) | <0.0001 | 1.52 (0.89-2.59) | 0.12 | NA | NA | NA | NA |
| Anti-6454 | 0.51 (0.42-0.63) | <0.0001 | 0.88 (0.49-1.60) | 0.68 | 1.27 (0.86-1.87) | 0.22 | NA | NA |
| Anti-A4U | 0.62 (0.48-0.79) | 0.0001 | 1.63 (1.06-2.53) | 0.03 | NA | NA | NA | NA |
| Anti-SAO75 | 0.54 (0.45-0.66) | <0.0001 | 1.00 (0.67-1.49) | 0.99 | NA | NA | NA | NA |
| Anti-NF54 (ICAM1/CD36) | 0.61 (0.48-0.78) | 0.0001 | 1.81 (1.07-3.03) | 0.03 | NA | NA | NA | NA |
| Antibody breadth | 0.33 (0.24-0.46) | <0.0001 | 0.23 (0.10-0.50) | 0.0002 | 0.47 (0.25-0.86) | 0.01 | 0.46 (0.32-0.67) | <0.0001 |
| Anti-PF3D7_1150400 | 0.83 (0.71-0.96) | 0.01 | 1.27 (0.69-2.32) | 0.44 | NA | NA | NA | NA |
| Anti-PF3D7_0425800 | 0.98 (0.79-1.22) | 0.85 | NA | NA | NA | NA | NA | NA |
| Binding inhibition | 0.58 (0.40-0.83) | 0.0028 | 0.71 (0.43-1.16) | 0.17 | NA | NA | NA | NA |
| Anti-Schizont | 0.13 (0.21-0.34) | <0.0001 | 0.31 (0.17-0.55) | 0.0001 | 0.36 (0.22-0.60) | 0.0001 | 0.40 (0.25-0.64) | 0.0001 |

Multivariable (restricted) : Stepwise regression including the factors indicated in the table.

NA: Indicates factors not included in the model


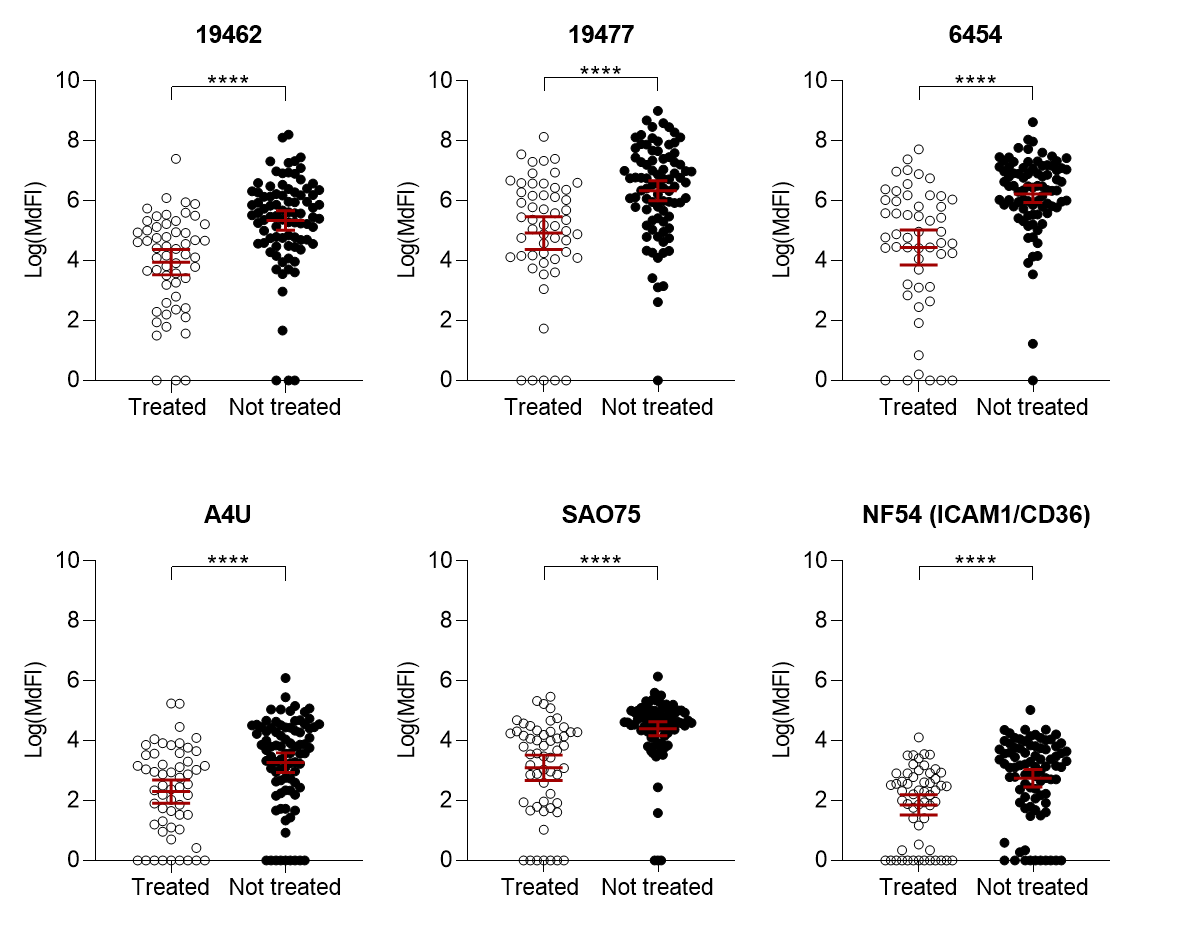


**Supplementary Figure 3: Anti-VSA antibodies and treatment outcome.** Comparison of anti-VSA IgGs at C-1 among individuals who developed symptoms or reached a parasite threshold for treatment during the study and those who did not. (P-values are indicated as follows * = 0<0.05, ** = <0.01 , *** = <0.001 and **** =<0.0001))


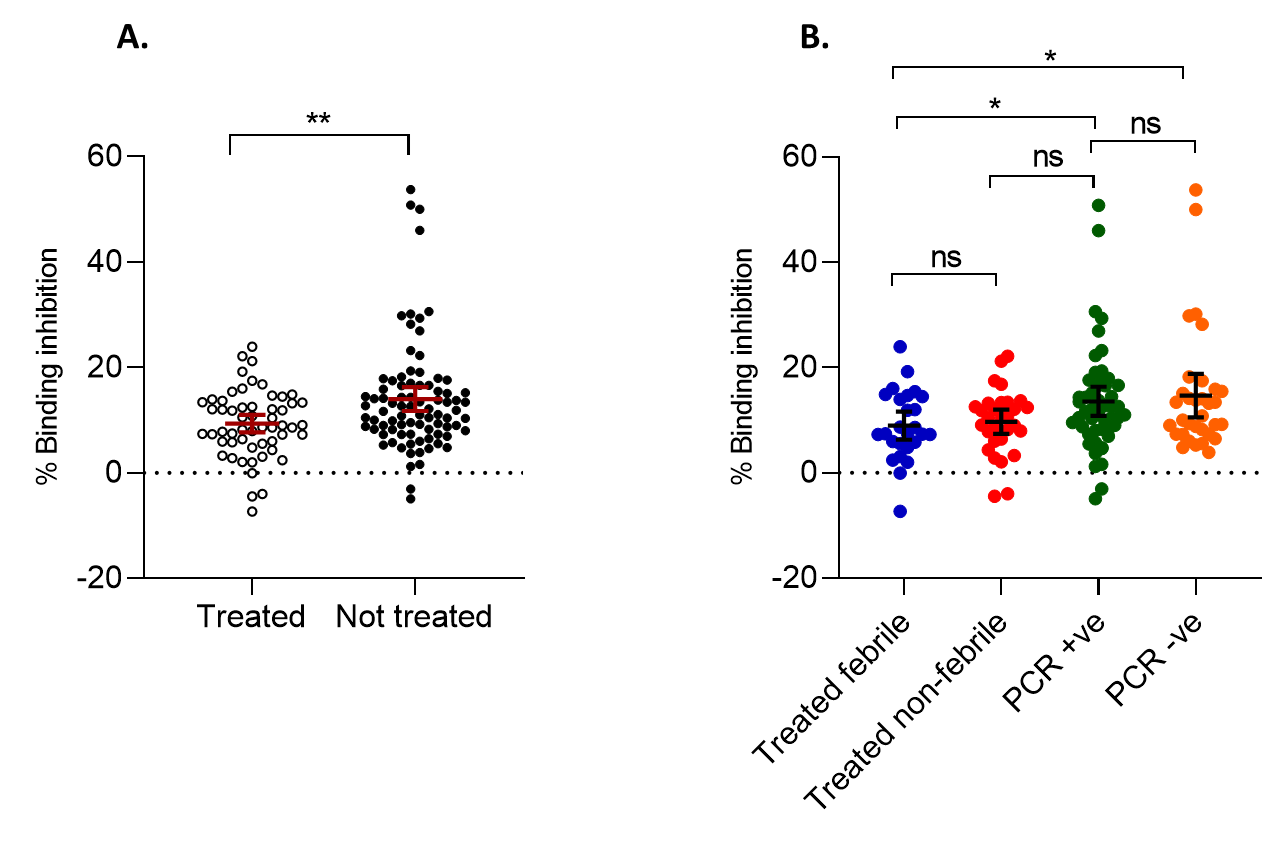


**Supplementary Figure 4:** Inhibition of PfEMP1-ICAM-1 binding stratified by CHMI outcome. Samples analysed were collected a day before the challenge (C-1). P-values are indicated as follows * = 0<0.05, ** = <0.01, ns=not significant.


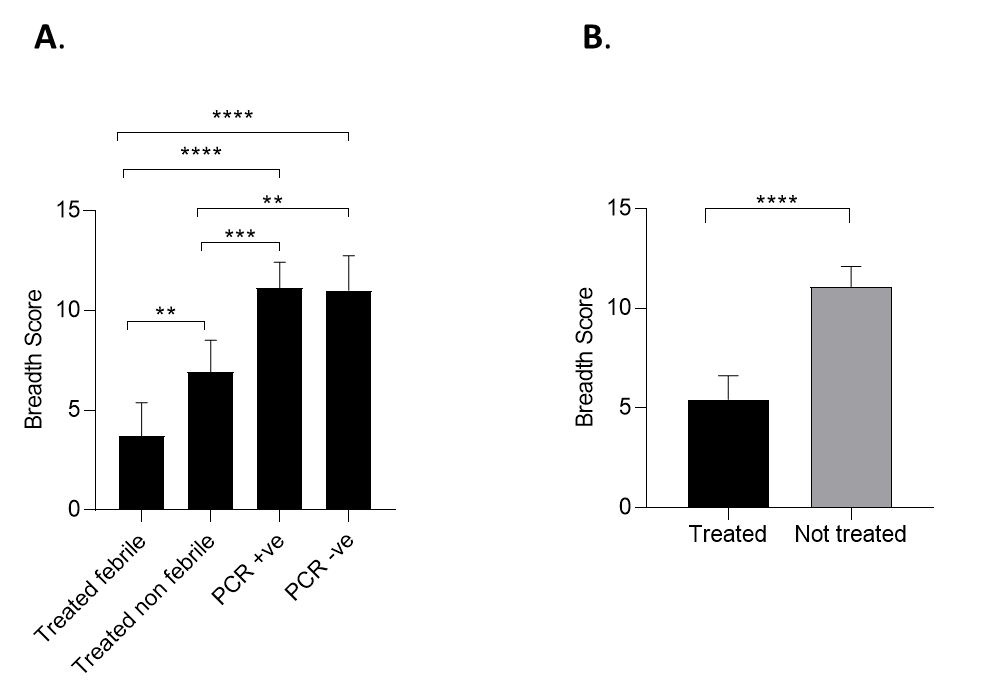


**Supplementary Figure 5:** Anti-VSA Antibodies breadth score among the CHMI outcome groups **(A)** and between treated and non-treated groups **(B).** P-values are indicated as follows * = 0<0.05, ** = <0.01, *** = <0.001, and **** = <0.0001). Samples analysed were collected a day before the challenge (C-1).


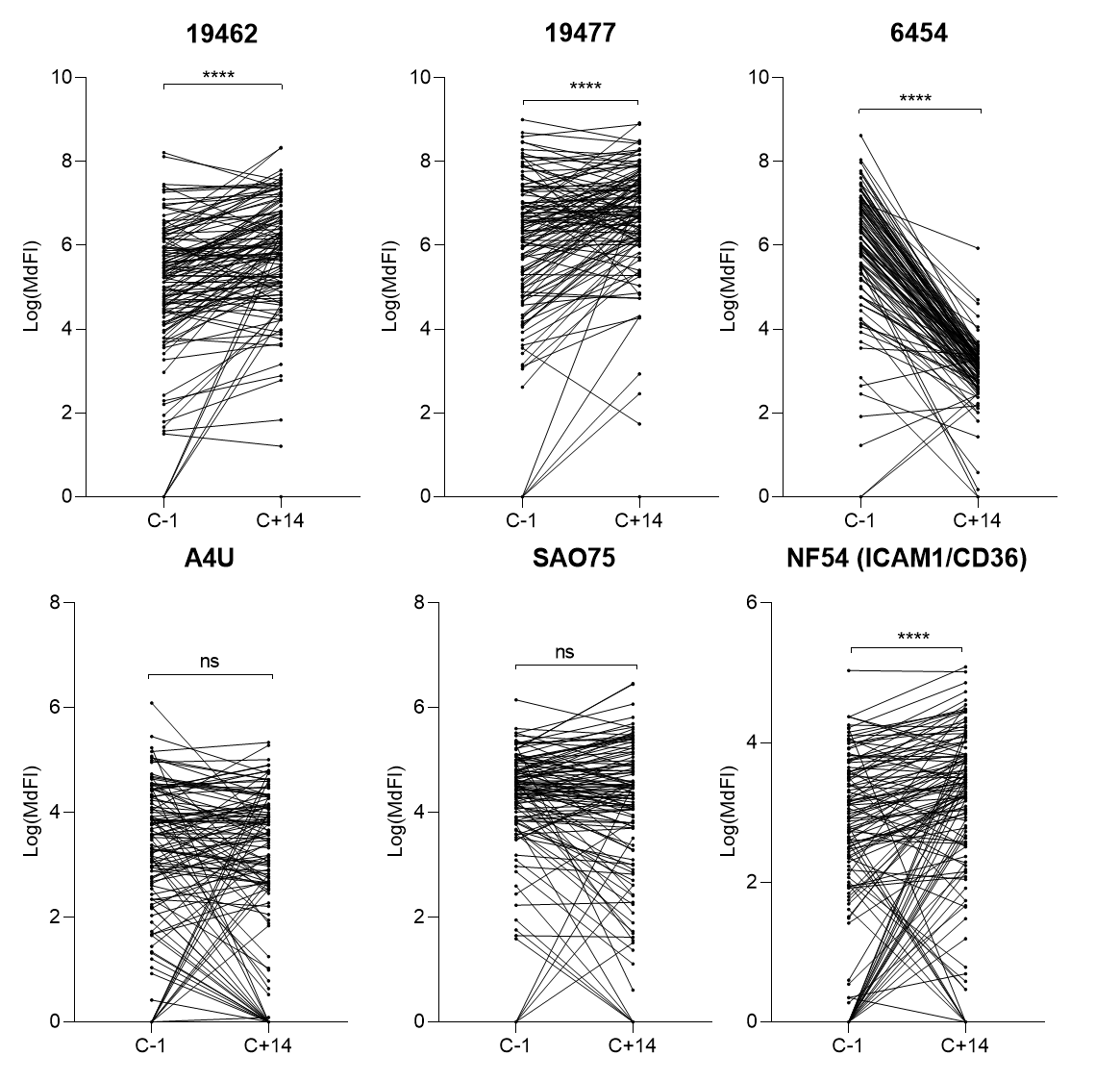


**Supplementary Figure 6:** Change in heterologous anti-VSA antibodies levels after the challenge. Comparison of anti-VSA antibody levels a day before (C-1) and 14 days after (C+14) the challenge. P value **** = <0.0001, ns= not significant
